# Regulation of microRNA during cardiomyocyte maturation in sheep

**DOI:** 10.1186/s12864-015-1693-z

**Published:** 2015-07-22

**Authors:** Janna L Morrison, Song Zhang, Ross L Tellam, Doug A Brooks, I Caroline McMillen, Enzo R Porrello, Kimberley J Botting

**Affiliations:** Early Origins of Adult Health Research Group, University of South Australia, Adelaide, SA Australia; Mechanisms in Cell Biology and Diseases Research Group, University of South Australia, Adelaide, SA Australia; Laboratory for Cardiac Regeneration, School of Biomedical Sciences, University of Queensland, St Lucia, QLD Australia; CSIRO Agriculture, CSIRO, Queensland Biosciences Precinct, St Lucia, QLD Australia

**Keywords:** Microarray, miR-133, miR-15 family, miR-590, miR-199a, Cardiomyocyte, Proliferation

## Abstract

**Background:**

There is a limited capacity to repair damage in the mammalian heart after birth, which is primarily due to the inability of cardiomyocytes to proliferate after birth. This is in contrast to zebrafish and salamander, in which cardiomyocytes retain the ability to proliferate throughout life and can regenerate their heart after significant damage. Recent studies in zebrafish and rodents implicate microRNA (miRNA) in the regulation of genes responsible for cardiac cell cycle progression and regeneration, in particular, miR-133a, the miR-15 family, miR-199a and miR-590. However, the significance of these miRNA and miRNA in general in the regulation of cardiomyocyte proliferation in large mammals, including humans, where the timing of heart development relative to birth is very different than in rodents, is unclear. To determine the involvement of miRNA in the down-regulation of cardiomyocyte proliferation occurring before birth in large mammals, we investigated miRNA and target gene expression in sheep hearts before and after birth. The experimental approach included targeted transcriptional profiling of miRNA and target mRNA previously identified in rodent studies as well as genome-wide miRNA profiling using microarrays.

**Results:**

The cardiac expression of miR-133a increased and its target gene *IGF1R* decreased with increasing age, reaching their respective maximum and minimum abundance when the majority of ovine cardiomyocytes were quiescent. The expression of the miR-15 family members was variable with age, however, four of their target genes decreased with age. These latter profiles are inconsistent with the direct involvement of this family of miRNA in cardiomyocyte quiescence in late gestation sheep. The expression patterns of ‘pro-proliferative’ miR-199a and miR-590 were also inconsistent with their involvement in cardiomyocyte quiescence. Consequently, miRNA microarray analysis was undertaken, which identified six discrete clusters of miRNA with characteristic developmental profiles. The functions of predicted target genes for the miRNA in four of the six clusters were enriched for aspects of cell division and regulation of cell proliferation suggesting a potential role of these miRNA in regulating cardiomyocyte proliferation.

**Conclusion:**

The results of this study show that the expression of miR-133a and one of its target genes is consistent with it being involved in the suppression of cardiomyocyte proliferation, which occurs across the last third of gestation in sheep. The expression patterns of the miR-15 family, miR-199a and miR-590 were inconsistent with direct involvement in the regulation cardiomyocyte proliferation in sheep, despite studies in rodents demonstrating that their manipulation can influence the degree of cardiomyocyte proliferation. miRNA microarray analysis suggests a coordinated and potentially more complex role of multiple miRNA in the regulation of cardiomyocyte quiescence and highlights significant differences between species that may reflect their substantial differences in the timing of this developmental process.

**Electronic supplementary material:**

The online version of this article (doi:10.1186/s12864-015-1693-z) contains supplementary material, which is available to authorized users.

## Background

From late gestation, the majority of human cardiomyocytes cease proliferating due to either an absence of karyokinesis and/or cytokinesis [1–3]. Consequently, and in contrast to the neonatal mammalian heart, the adult heart has limited capacity to regenerate cardiomyocytes that may be lost due to damage or aging [4–6]. This is dissimilar to zebrafish, whose cardiomyocytes retain the ability to proliferate throughout life and are therefore able to regenerate the heart after significant damage [7, 8]. The majority of regenerated zebrafish and neonatal mouse cardiomyocytes originate from existing cardiomyocytes rather than cardiac stem cells [4, 9–12] and are less differentiated and have less contractile organisation compared to those from un-injured myocardium [4, 9]. Recent evidence suggests that microRNA (miRNA) regulate cardiomyocyte proliferation and can induce reversion of adult cardiomyocytes to a pro-proliferative state [13].

miRNA are small (~22 nucleotide) non-coding RNAs that are often evolutionarily conserved between species. miRNA originate from longer gene transcripts that form hairpin loops, which are cleaved by the RNAase enzymes, Drosher and Dicer, to generate mature miRNA. Once bound to the Argonaut protein, miRNA guide the RNA-induced silencing complex (RISC), sometimes referred to as miRNP (miRNA-ribonucleoprotein complex), to mRNA targets and subsequently regulate the expression of genes by suppressing translation and/or initiating the degradation of the mRNA. There are in excess of a thousand miRNA encoded by the human genome and each miRNA can inhibit multiple genes, thereby potentially allowing the regulation of functional or developmental pathways (for review see [14]).

A comparison of miRNA expression in regenerated versus un-injured zebrafish myocardium identified miR-133 as being specifically down-regulated during the period of cardiomyocyte proliferation and regeneration [7]. There are three genomic loci producing miR-133 with only miR-133a-1 and miR-133a-2 being expressed in the heart [15]. miR-133 is one of the most abundant cardiac miRNA [16] and is essential for normal cardiogenesis in mice, through regulation of serum response factor (*SRF*) [GenBank: NM_020493] dependent transcription [17]. Interestingly, *SRF* together with Cyclin D2 (*CCND2*) [GenBank: NM_009829] was associated with increased cardiomyocyte proliferation in miR-133a-1/miR-133a-2 double-knockout mice [17]. miR-133a also regulates Connexin-43 (*GJA1*) [GenBank: NM_131038] [7], phosphoglycerate mutase (*PGAM1*) [GenBank: NM_023418] [18] and connective tissue growth factor (*CTGF*) [GenBank: NM_010217] [19–21] in regenerative and proliferative cardiomyocytes. miR-133a has also been implicated as an inhibitor of vascular smooth muscle [22] and ovarian cancer [23] cell proliferation, through the inhibition of insulin-like growth factor receptor 1 (*IGF-1R*) [GenBank: NM_010513], which is a potent stimulator of cardiomyocyte proliferation [24, 25].

Murine cardiomyocytes transition from a proliferative to a quiescent state from days 4 to 10 after birth [26]. Comparisons of miRNA expression across this period identified miR-195, a member of the miR-15 family, as the most up-regulated miRNA and therefore possibly implicated in the inhibition of cardiomyocyte proliferation [27]. Furthermore, across the period of diminished proliferation in mice, the mRNA expression was reduced for both conserved and non-conserved target genes of the miR-15 family, such as Checkpoint kinase 1 (*Chek1*) [GenBank: NM_007691], Cyclin dependent kinase-1 (*Cdk1*/*Cdc2a*) [GenBank: NM_007659], Survivin/Baculoviral inhibitor of apoptosis repeat-containing *5* (*Birc5*) [GenBank: NM_001012273] and Sperm-associated antigen 5 (*Spag5*) [GenBank: NM_017407]. Furthermore, inhibiting the miR-15 family *in vivo* increased the number of mitotic cardiomyocytes in mouse [12, 27].

Both miR-133a and the miR-15 family are associated with inhibition of cardiomyocyte proliferation and myocardial regeneration, however, there are also miRNA that promote proliferation. miR-199a and miR-590 are involved in promoting proliferation in rodents [13]. In addition, treating adult rat cardiomyocytes with mimics for miR-199a and miR-590 promotes cell cycle re-entry and promotion of cardiac regeneration *in vivo* [13]. Using short interfering RNA that were specific for each gene target of miR-199a and miR-590, the knockdown of 43 genes increased the percentage of cardiomyocytes undergoing DNA synthesis by approximately 2-fold. Of the genes that were associated with the up-regulation of DNA synthesis, three genes (Chloride intracellular channel protein 5 (*Clic5*) [GenBank: NM_172621]*,* Homeodomain-only protein (*HopX*) [GenBank: NM_001159900] and Homer protein homolog 1 (*Homer1*) [GenBank: NM_001284189]) were targets of both miR-199a and miR-590 and are therefore believed to be important regulators of cell cycle activity in cardiomyocytes.

Mouse and rat cardiomyocytes become quiescent in postnatal life and this process is complete by day 10 [26] and 12 [28], respectively. The timing of quiescence in rodents corresponds to a period just after birth when there is significant cardiac remodelling triggered by changes in the circulation due to air breathing and the switch in cardiomyocyte metabolism from predominantly glycolysis *in utero* to fatty acid oxidation in postnatal life [29]. Hence, changes in miRNA and target gene expression during this period in rodents are difficult to interpret due to metabolic changes occurring with developmental processes. Quiescent human cardiomyocytes have been identified from as early as 0.8 of gestation [30] and the process is generally near complete by birth, however, recent studies demonstrate that a very low level cardiomyocyte proliferation may extend to 20 years of age [31]. The *in utero* transition to quiescence in humans is similarly timed to sheep, a species where cardiomyocytes become quiescent due to binucleation from 0.75 of gestation [32] and the percentage of cardiomyocytes in the cell cycle decreases from 7 % at 110 days gestation to 1 % close to birth [33]. The current investigation aimed to gain greater insight into the regulation of human cardiomyocyte proliferation by determining the expression of specific miRNA and their target genes, from the aforementioned zebrafish and murine studies, in sheep myocardium across late gestation and early postnatal life. Through the use of a miRNA microarray, we further aimed to determine the expression of all miRNA across this developmental window, thereby isolating the miRNA that may regulate cardiomyocyte quiescence from those associated with the major physiological and biochemical changes that occur just after birth.

## Results

### Body and heart weight measurements

Body weight and heart weight data were collected at each time point and, as expected, increased with age (*P* < 0.05; Table 1). Heart and body growth patterns were in unison until 5 days of age, such that heart weight relative to body weight was maintained, however, at 21 days of age and furthermore at 173 days of age, heart weight relative to body weight decreased (*P* < 0.05).

**Table 1:** Fetal body and heart weight measurements

	Body weight (kg)	Heart weight (g)	Heart weight/Body weight (g/kg)
F91	0.67 ± 0.02^a^	4.41 ± 0.86^a^	6.67 ± 1.32^c^
F120	2.38 ± 0.14^a^	17.42 ± 1.11^a^	7.36 ± 0.30^c^
F140	5.18 ± 0.17^b^	38.5 ± 2.25^b^	7.46 ± 0.51^c^
P5	5.46 ± 0.16^b^	43.47 ± 2.57^b^	7.93 ± 0.29^c^
P21	13.21 ± 0.31^c^	81.38 ± 3.26^c^	6.19 ± 0.33^b^
P173	42.39 ± 1.38^d^	196.09 ± 6.94^d^	4.63 ± 0.07^a^

*F* Fetal age, *P* Postnatal age in days; Values are mean ± SEM. Values with a different letter signify that age points are significantly different from each other. *P* <0.05 was considered significant

### Expression of miRNA involved in regulation of cardiac proliferation and their target genes

Two complementary experimental strategies were used to profile ovine miRNA and their target genes over the cardiac developmental period from 91 days of gestation to 173 days of age. First, qRT-PCR was used to measure the expression of specific ovine miRNA and target genes associated with rodent and zebrafish cardiomyocyte quiescence. Second, for comprehensive analysis a miRNA microarray was used to profile over 3000 probes representing miRNA from several mammalian species, including sheep and a bioinformatics approach was undertaken to predict enriched functions associated with clusters of developmentally regulated miRNA.

### Developmental profiling of rodent and zebrafish miRNA by qRT-PCR

The ovine cardiac expression of three sets of miRNA, which are highly conserved across species, and their target genes that were previously associated with regulation of cardiomyocyte proliferation in rodent and zebrafish [7, 12, 13] were measured by qRT-PCR at each developmental age to investigate their roles in prenatal cardiomyocyte quiescence in a larger mammal, sheep, characterised by quiescence of cardiomyocytes in late gestation.

#### miR-133a

**Fig. 1:**
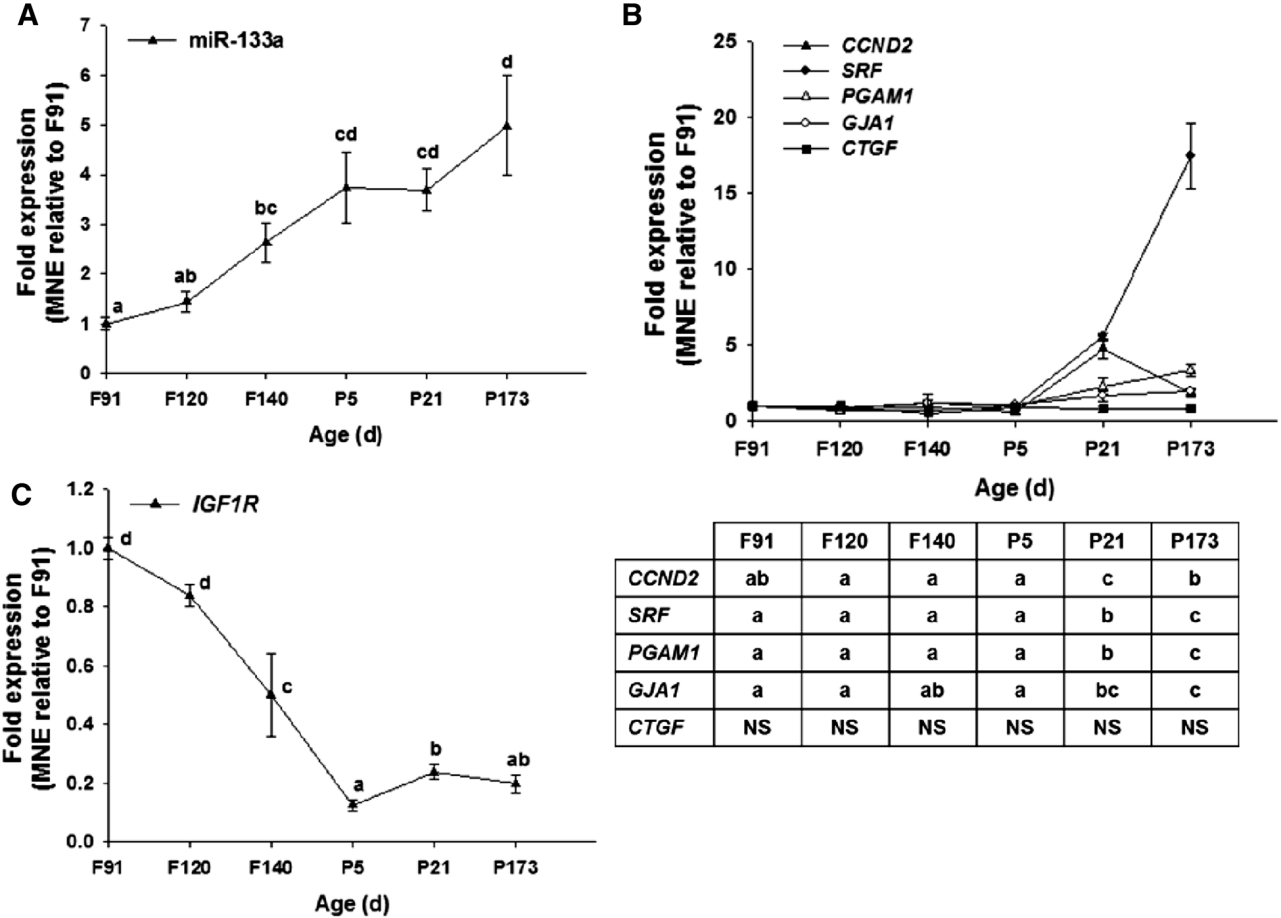
Expression of miR-133a and its target mRNAs during heart development. miR-133a increased with age (**a**) and the expression of its target genes, *PGAM1*, *GJA1, SRF* and *CCND2* increased between 5 and 21 days of age (**b**) and *IGF1R* decreases with age (**c**). Data are expressed relative to 91 days gestation. *F* Fetal, *P* Postnatal, *d* age in days, *NS* not significant. Values with a different letter signify that age points are significantly different from each other. *P* <0.05 was considered significant

The expression of miR-133a increased with age and reached its maximum expression by 5 days of age (*P* < 0.05; Fig. 1a). Counterintuitively, the expression of the miR-133a gene targets that promote cardiomyocyte proliferation in rodents, *CCND2*, *SRF*, *PGAM1* and *GJA1,* had their highest expression after birth (*P* < 0.05; Fig. 1b), when the majority of sheep cardiomyocytes were binucleated and quiescent. Furthermore, the expression of miR-133a gene target *CTFG* did not change with age (Fig. 1b). These results suggest that these genes are not directly regulated by miR-133a in sheep or are subject to more complex and overriding developmental regulators. The result also indicates that these genes are unlikely to be directly involved in ovine cardiomyocyte quiescence in sheep. The increased expression of miR-133a, however, coincided with decreased expression of *IGF1R,* a known target for this miRNA [22, 23] (*P* < 0.05; Fig. 1c).

#### miR-15 family

**Fig. 2:**
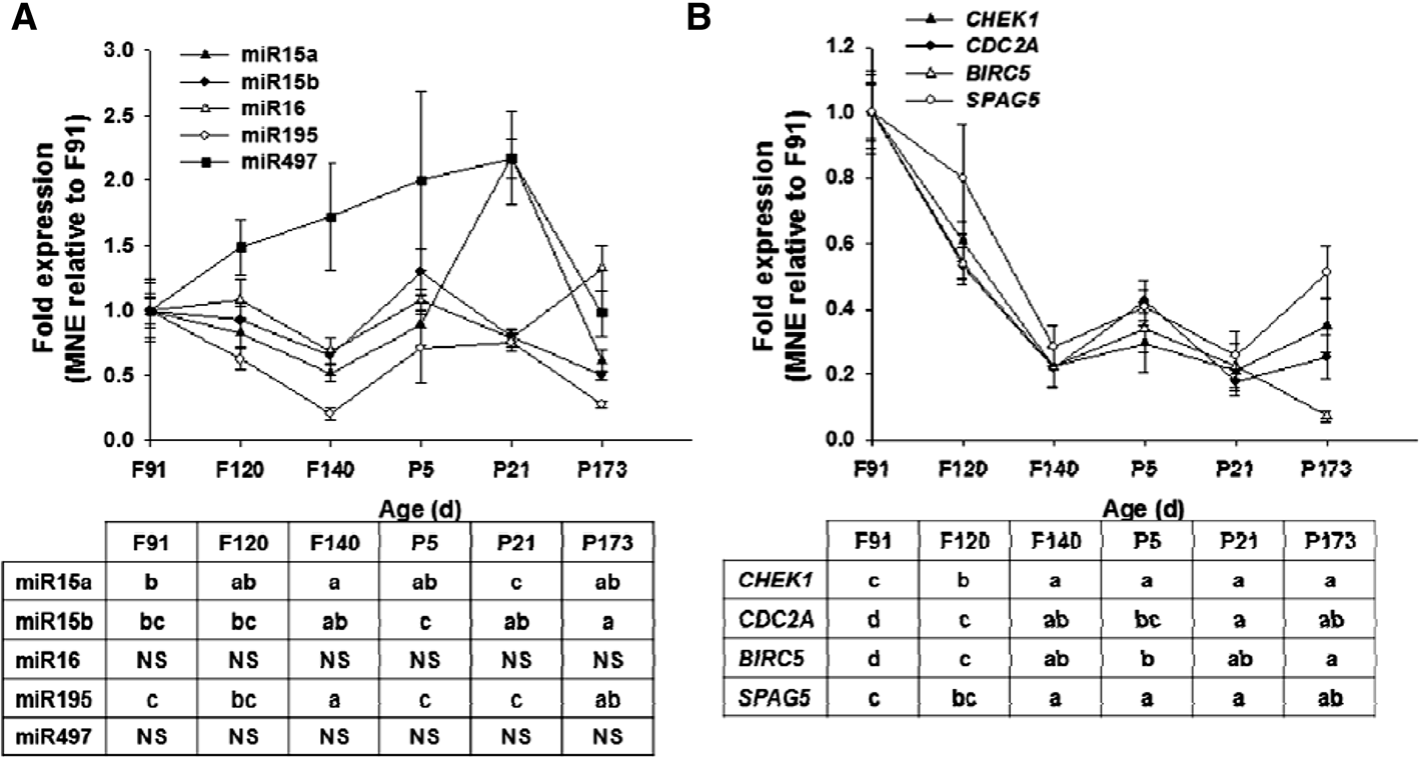
Expression of miR-15 family members and their target mRNA during heart development. The expression of miR-15 family members is variable with age (**a**), however, the expression of their target genes *CHEK1*, *CDC2A*, *BIRC5* and *SPAG5* decreased with age (**b**). Data are expressed relative to 91 days gestation; *F* Fetal, *P* Postnatal, *d* age in days, *NS* not significant. Values with a different letter signify that age points are significantly different from each other. *P* <0.05 was considered significant

The miR-15 family contains five miRNA, miR-15a, miR-15b, miR-16, miR-195 and miR-497. Ovine cardiac expression of miR-15a expression was lowest at 140 days gestation and highest at 21 days of age (*P* < 0.05), the expression of miR-15b was lowest at 173 days of age and highest at 5 days of age (*P* < 0.05) and miR-195 expression was lowest at 140 days gestation and highest at 91 days gestation, 5 and 21 days of age (*P* < 0.05). The expression of both miR-16 and miR-497 did not change with age (Fig. 2a). Despite the variable expression patterns of members of the miR-15 family, the expression of their target mRNAs (*CHEK1*, *CDC2A*, *BIRC5* and *SPAG5)* decreased with increasing age, reaching a minimum and then plateauing at 140 days gestation (*P* < 0.05; Fig. 2b). Given that these genes are involved in cell cycle progression and mitosis [34–37], these results suggest that the decreased expression of these genes may be involved in ovine cardiomyocyte quiescence, however, the miR-15 family may not be the principal developmental regulator of these genes or ovine cardiomyocyte quiescence.

#### miR-199a and miR-590

**Fig. 3:**
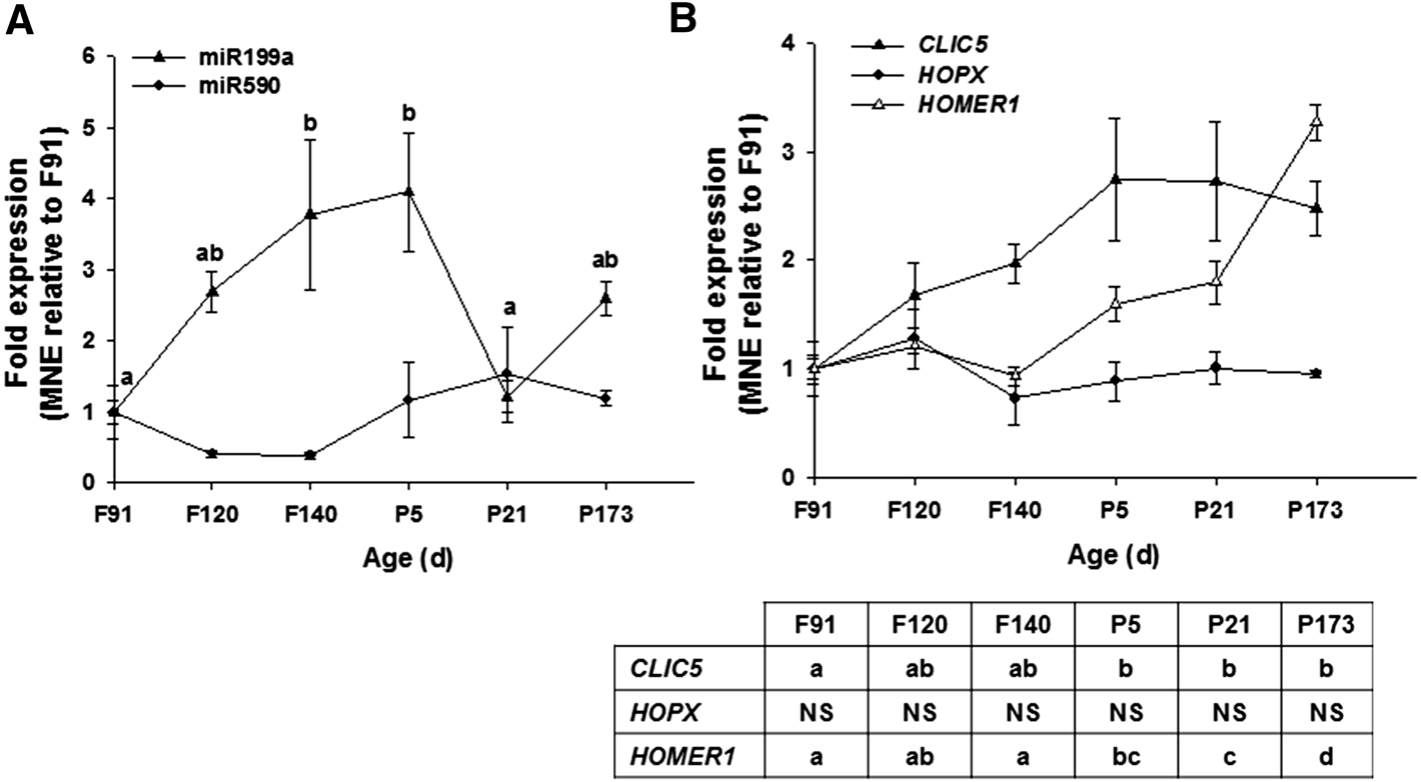
Expression of pro-proliferative miR, mir-199a and miR-590, and their target mRNA during heart development. miR-199a increases with age, but is then reduced to the level seen at 91 days gestation by 21 days of age (**a**). The expression of *CLIC5* and *HOMER1* is increased in postnatal life (**b**). Data are expressed relative to 91 days gestation; F, Fetal; P, Postnatal; d, age in days. Values with a different letter signify that age points are significantly different from each other. *P* <0.05 was considered significant

The expression of miR-199a peaked around birth, but then decreased by 21 days of age to the level seen at 91 days gestation (*P* < 0.05; Fig. 3a). The expression of miR-590, however, did not change with age (Fig. 3a). The expression of *HOPX*, a target of both miR-199a and miR-590 [13], did not change with age (Fig. 2b), however, the expression of *CLIC5* and *HOMER1* increased with age and reached their maximum expression by 5 days and 173 days of age, respectively (Fig. 2b). These data suggest that miR-199a and miR-590 miRNA and their target genes may not be directly involved in ovine cardiomyocyte quiescence.

### miRNA microarray analysis

**Fig. 4:**
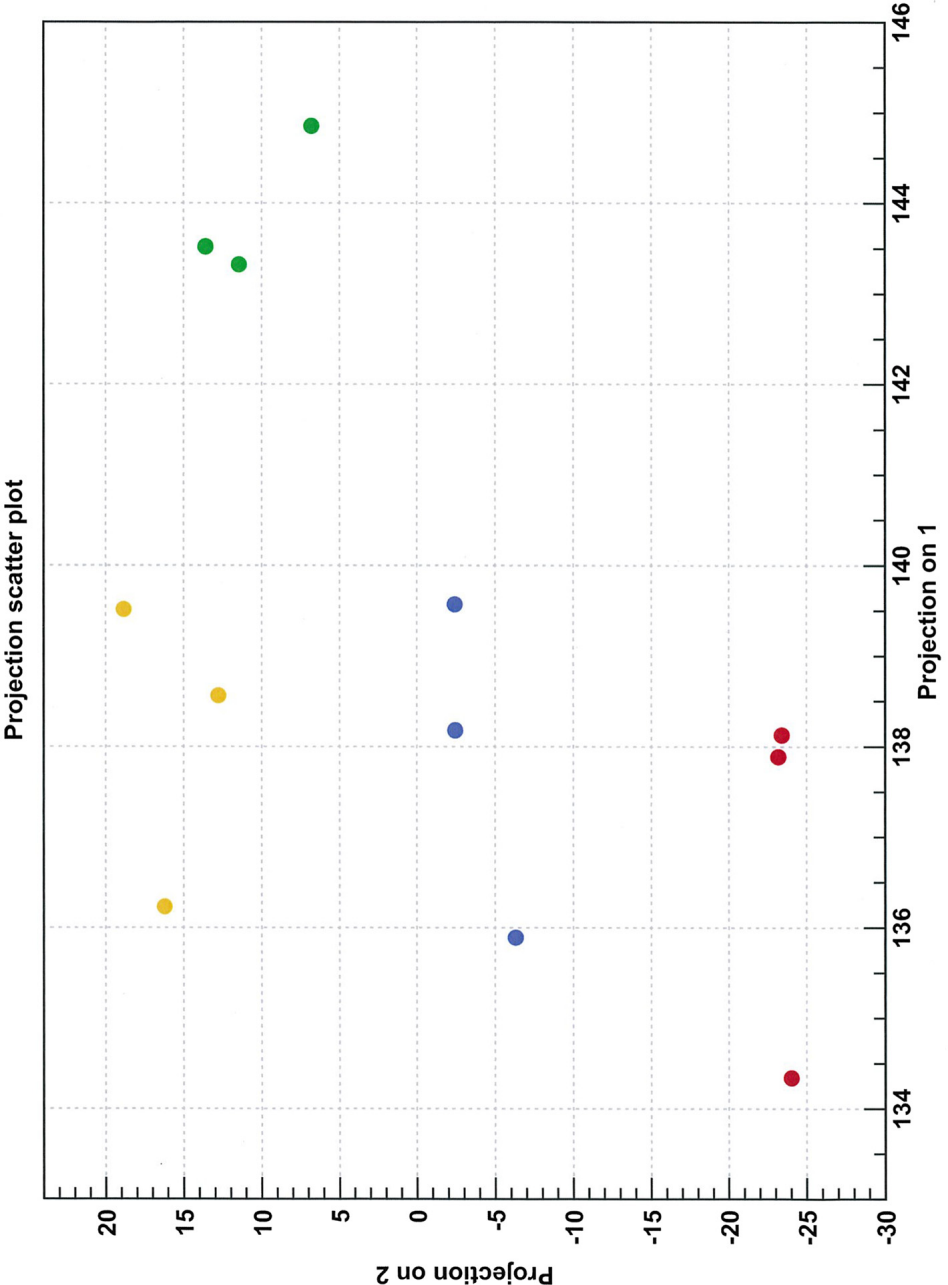
Principal Components Analysis of the miRNA microarray data. The data represented in a PCA plot show separation of the four developmental age groups and clustering of the biological replicates within these groups. Red, Fetal 91 days gestation; Green, Fetal 140 days gestation; blue, Postnatal 5 days; yellow, Postnatal 173 days

**Fig. 5:**
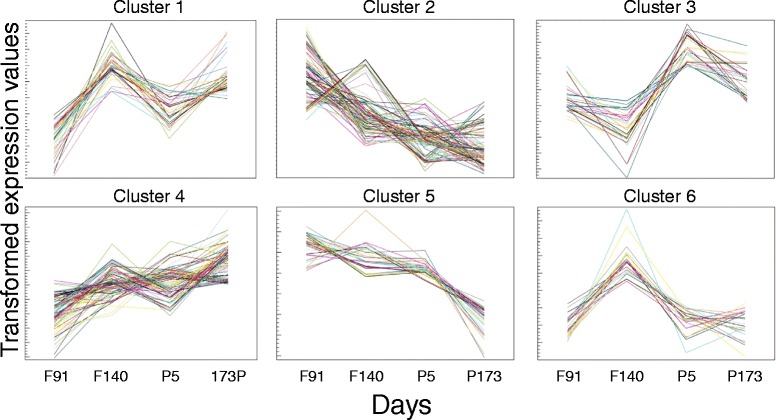
k-means clustering of the differentially expressed miRNA. miRNA were grouped into co-expression clusters using k-means clustering. A summary of non-redundant miRNA present in each k-means cluster can be found in Table 2. *F* Fetal, *P* Postnatal, *d* age in days

The targeted profiling of miRNA identified in rodent and zebrafish investigations showed limited correspondence with the developmental program of ovine cardiomyocyte quiescence occurring during late gestation in sheep. Consequently, miRNA microarrays were used to investigate age related changes in cardiac miRNA in a subset of the animals (*n* = 3 at each of Fetal (F) 91, F140, Postnatal (P) 5 and P173). This approach complements the targeted miRNA approach and has the potential to identify additional miRNA and possibly broader functional themes associated with cardiac development in sheep. A custom designed miRNA microarray that incorporated multiple replicates of 3,098 unique probes for sheep and other mammalian miRNA was used. Technical limitations of the approach are listed in the Methods. A PCA analysis of the microarray data shows separation of the four developmental groups and clustering of the biological replicates within these groups (Fig. 4). The first component in the analysis explained 97 % of the variation. A total of 297 (9.6 %) differentially expressed probes (FDR corrected *P* ≤ 0.05) were identified, which included 14 anonymous probes and some redundant probes. The full analysis is presented in Additional file 1. The analysis clearly indicates that multiple miRNA are involved in cardiac development and age related functions, and suggests substantial complexity in the regulation of target gene expression orchestrating these developmental and physiological changes.

k-means clustering of the differentially expressed miRNA separated the developmental data into six discrete clusters (Fig. 5). This analysis posits that miRNA within a cluster have functions pertaining to the specific developmental transition represented by the cluster profile. The largest clusters were 2 and 4 containing 88 and 91 probes, respectively (Additional file 1 and Table 2). The former cluster represented a progressive decline in overall signal with all developmental time points while the latter cluster represented a progressive increase of signal with development. Clusters 1, 3 and 6 (35, 28 and 23 probes, respectively) showed more complex developmental profiles that may reflect key developmental transitions in the myocardium and the indirect impacts of the major physiological and biochemical changes occurring after birth. In particular, these clusters are characterised by major transitions between the late gestation fetus at 140 days gestation and the lamb at 5 days after birth. Cluster 5 showed a profile characterised as a small continuous decline until 5 days after birth followed by a more substantial decline to 173 days after birth.

**Table 2:** Summary of nonredundant miRNA present in each k-means cluster

Cluster number	miRNA^a^
1	miR-29a-3p; miR-29b; miR-1-1-5p; miR-1-2-5p; miR-142-3p; miR-499b-3p; miR-101; mir-2284z-6-p3; miR-6412; mir-2284z-5-p5; miR-499-5p; miR-499; miR-499b-5p; miR-1; miR-451; miR-126a-5p; miR-6119-5p; miR-10b; miR-1-5p; **miR-195**; miR-140-5p; miR-29d-3p; miR-29a; miR-101c; miR-208b
2	mir-3431-p3; miR-433-3p; mir-3431-p3; mir-3431-p5; miR-503-5p; miR-324; miR-542-5p; miR-382-3p; miR-181a-2-3p; miR-3431; miR-324-3p; miR-362-5p; miR-362; miR-181b-5p; miR-181b; miR-106b-3p; mir-181b-2-p3; miR-204; **miR-199a**-5p; miR-484; miR-155; miR-20b; miR-1343-3p; miR-224; miR-132-3p; miR-106a; mir-99b-p5; miR-99b; miR-17-5p; miR-93; mir-466i-p5; miR-16a; miR-16b; **miR-16-5p**
3	miR-2137; mir-1285-p5; mir-6323-p5; PC-5p-51320_64; miR-1973; mir-1973-p3; miR-4792; mir-4485-p5; mir-940-p5; miR-4448; miR-671-5p; mir-2487-p5; miR-1835; mir-4419a-p5; mir-1246-p5; mir-218-1-p5; miR-122; mir-466b-2-p3; mir-378f-p5; mir-4426-p3
4	miR-145; miR-133b; miR-378; **miR-133a**; miR-378c; miR-378b; miR-133; miR-378d; miR-24; miR-24-3p; mir-7641-1-p5; miR-133b-3p; miR-378f; miR-378i; mir-7641-1-p3; miR-27b; miR-133c; miR-1260b; miR-1260; miR-185; miR-708; miR-30b-3p; miR-7977; mir-5100-p5; miR-5100; mir-708-p5; miR-4454; miR-486; miR-30c-1-3p; miR-150; mir-4454-p5; mir-5100-p3; miR-1386; mir-6240-p3; miR-345-5p; mir-4454-p3; miR-193b; miR-22-3p; miR-1386; mir-2285 m-3-p5; miR-193a-3p; miR-486-5p; miR-22-5p; **miR-497**; mir-2285 k-3-p5; miR-29a; miR-34
5	miR-543-3p; miR-3959-3p; miR-3958-3p; miR-154b; miR-154b-5p; miR-493-5p; miR-432; miR-485-3p; miR-380-3p; miR-324-5p; miR-154a-3p; mir-324-p5; miR-379-3p; miR-382-5p; miR-411b-3p; miR-409-3p; miR-411a-3p; miR-379-5p; miR-487b-3p; miR-329a-3p; miR-329b-3p; **miR-15b**; miR-127; mir-15b-p3; miR-15b-5p; miR-130a; miR-214; mir-376d-p3; miR-410
6	miR-542-3p; mir-2284f-p3; miR-322-5p; miR-376c-3p; miR-369-3p; miR-376d; miR-376e-3p; let-7a-p3; miR-424-5p; miR-218a; miR-144-3p; miR-20a-5p; miR-21; miR-21a-5p; miR-374a; miR-19b; miR-301a; miR-450b; miR-499-3p; miR-208b

^a^All species identifiers were removed. Fourteen anonymous probes beginning with the designation ‘PC’ were excluded, but can be found with species identifiers in Additional file 1.^b^ miRNA included in the targeted qRT-PCR investigatation highlighted in bold.

In general, the patterns of the miRNA measured by qRT-PCR were consistent with the cluster profiles. miR-133a was present in Cluster 4 and displayed the same pattern of expression in both qRT-PCR and microarray analysis. miR-15 family members miR-15b, miR-16, miR-195 and miR-497 were identified in Clusters 5, 2, 1 and 4, respectively. miR-199a was identified in Cluster 2 and the expression pattern of miR-590 did not allow its inclusion in any cluster.

**Fig. 6:**
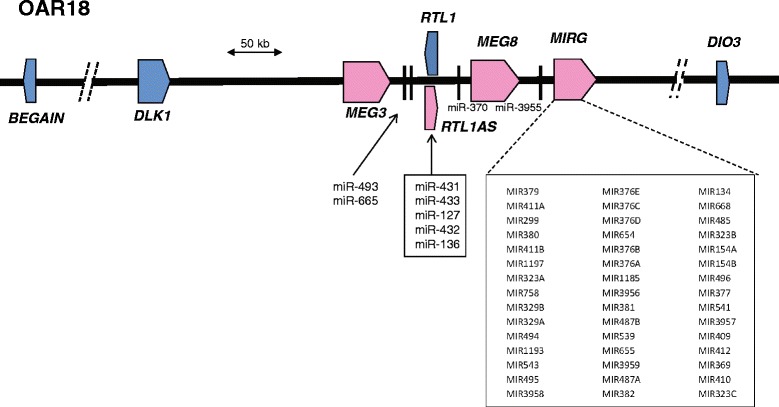
Schematic representation of an imprinted genomic segment on the telomeric arm of ovine chromosome 18. The genomic region highlights multiple miRNA many of which were identified as differentially expressed in Cluster 5. Blue, paternally expressed protein encoding genes. Pink, maternally expressed noncoding RNA. The central region contains a number of maternally expressed miRNA including a large cluster designated *MIRG. MEG8* contains a large cluster of snoRNA while *MEG3* is a long noncoding RNA. *RTL1AS* transcribes a noncoding RNA containing five miRNA, which are known to cause RISC mediated cleavage of the sense protein encoding gene *RTL1*. All maternally expressed genes are transcribed in the same direction

miRNA are often genomically positioned in groups on chromosomes and we therefore investigated whether any of the miRNA clusters were associated with specific chromosomal regions. By mapping the miRNA to ovine chromosomes (UCSC Genome Browser; OAR_c3.1 sheep assembly [38]), we determined that Cluster 5 was strongly overrepresented by miRNA (83 % of all miRNA in Cluster 5) associated with a large and complex group of miRNA in a 250 kb region located toward the telomeric end of OAR18 (Fig. 6). The broader 1.1 Mb region encompassing the protein encoding genes *BEGAIN* to *DIO3* is imprinted and the miRNA within this region are maternally expressed [39–41]. The biological and molecular roles of these miRNA are unclear.

**Fig. 7:**
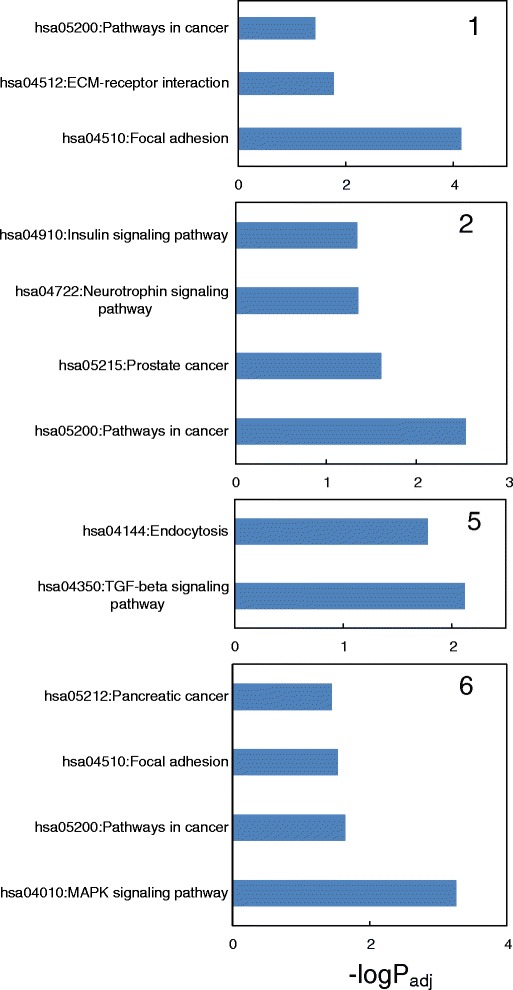
Representations of enriched KEGG pathways for predicted target genes associated with each k-means cluster. DAVID was used to identify enriched KEGG pathways [43]. An FDR adjusted *P-value* <0.05 was considered significant. The top ranked five pathways are shown. Clusters 3 and 4 did not produce any significantly enriched KEGG pathways. Each cluster is identified by a number. FDR corrected *P*-values are shown on a –log_10_ scale (significance cut-off >1.3). Full data for enriched KEGG pathways and the three GO categories Biological Process, Cellular Component and Molecular Function are listed in Additional file 2

To identify putative mRNA targets for the miRNA, a non-redundant set of miRNA corresponding to each k-means cluster (filtered for removal of low expression miRNA) was used for mRNA target prediction using MiRWalk. The strategy undertaken was highly conservative as MiRWalk was chosen to include a consensus of at least seven of ten different miRNA target prediction methods [42]. The numbers of unique target mRNAs for the six miRNA clusters were 1050, 1034, 914, 963, 989 and 1292, respectively. Enriched Gene Ontology (GO) terms and KEGG pathways were then identified using DAVID [43] (Fig. 7; FDR corrected *P* < 0.05 and limited to a maximum of the five top ranked pathways). The full KEGG pathway analysis and the enriched GO categories Biological Process, Cellular Component and Molecular Function for each cluster are listed in Additional file 2.

Cluster 1 was strongly associated with an extracellular matrix theme in the enriched KEGG pathway and GO Cellular Component (CC) analyses. There was also a theme related to regulation of transcription revealed by the GO Biological Process (BP) and Molecular Function (MF) categories. KEGG pathway analysis for Cluster 2 showed enrichment for *proliferative activity* and *insulin signalling* while *regulation of transcription* was a common theme in the GO BP and MF analyses. GO BP showed enrichment for aspects of macromolecule metabolism and biosynthesis. Cluster 3 showed enrichment for *protein transport* (GO BP) and *synapse* (GO CC), the latter possibly indicative of innervation changes. Cluster 4 was associated with *regulation of transcription* (GO BP and MF), *regulation of biosynthetic processes* (GO BP) and *neuron projection* (GO CC). Enrichment for the *TGFβ pathway* was linked with Cluster 5 as well as regulation of *phosphate metabolic processes* (GO BP), *Golgi* (GO CC) and *regulation of transcription* (GO MF). Cluster 6 showed mixed themes including *MAPK signalling* and *extracellular matrix* (KEGG), *regulation of transcription* (GO BP and MF) and *Golgi* (GO CC). Importantly, Clusters 1, 2 and 6 contained enriched pathways that have been associated with “*cancer*”, which likely reflect aspects of cell proliferation, while Clusters 1, 2 and 4–6 contained enrichment for terms associated with *transcription factor regulation*, which also may reflect transcriptional regulation of cardiomyocyte proliferative activity.

## Discussion

This study is the first to analyse the expression of miRNA in a mammalian model (sheep) where, as in humans, cardiomyocyte quiescence occurs *in utero.* The current study highlights species specific differences and some similarities in the expression patterns of specific miRNA previously implicated in the regulation of cardiomyocyte proliferation and regeneration in rodents and zebrafish. The interpretation of miRNA and target gene developmental profiles in different species may be complex. First, the sheep and rodent models of cardiac development are inherently different in terms of the timing of cardiomyocyte quiescence and moreover the latter models are potentially confounded by superimposition of developmental changes with large scale postnatal metabolic changes and cardiomyocyte hypertrophy. Second, some miRNA target genes may vary in the sequence of their potential miRNA recognition sites leading to changes in the binding affinity and stoichiometry of miRNA with target genes. This could generate altered molecular processes regulating similar developmental processes in a species specific manner. Third, changes in miRNA abundance may impact other related miRNA through direct competition for target mRNA. The use of a miRNA microarray in combination with targeted analyses in the sheep model has revealed enrichment for clusters of miRNA belonging to functional categories possibly associated with cardiomyocyte proliferation/quiescence such as ‘*proliferative activity’* and ‘*cancer’* (which is characterised by cell proliferation) and provided broad insight into the program of cardiac development in a large mammal where cardiomyocyte quiescence occurs during late gestation, similar to human and unlike rodents and zebrafish.

### Identification of miRNA with expression profiles consistent with involvement in inhibition of cardiomyocyte proliferation in sheep

Cardiomyocyte quiescence is observed in sheep from 0.75 of gestation [32] and only ~1 % of cardiomyocytes are in the cell cycle near term [33]. miRNA that are key inhibitors of cardiomyocyte proliferation would likely fall predominantly into miRNA Cluster 4 or possibly 1 and 3, since their expression patterns increase with age. Consistent with this view, miRNA implicated in rodent cardiomyocyte quiescence, miR-133a, miR-497 and miR-195, were identified in Clusters 4, 4 and 1, respectively, from the miRNA microarray analysis. In particular, miR-133a, the highly abundant cardiac miRNA implicated in regulation of cardiomyocyte proliferation in zebrafish [7] and mice [17], was confirmed by qRT-PCR and microarray analysis as increasing with developmental age in sheep. The role of miR-133a as a suppressor of cardiomyocyte proliferation is supported by both miR-133a double-knockout (miR-133a-1/miR-133a-2) mouse studies showing that miR-133a is essential for regulating cardiomyocyte proliferation and normal heart development [17], and zebrafish studies, where miR-133a was significantly reduced in regenerative/proliferative cardiac tissue [7]. The increased abundance of miR-133a coincided with the decreased expression of one of its gene targets, *IGF1R*. The IGF1R signalling pathway is an important regulator of cardiac development [25] and stimulates cardiomyocyte proliferation [24]. The current investigation is the first to highlight a potential link between miR-133a and *IGF1R* in the regulation of cardiomyocyte proliferation in a large mammal. However, the expression of other miR-133a target genes (identified in other species) that are pro-proliferative [7, 17–20], either increased in postnatal life (*SRF*, *CCND2*, *PGAM1* and *GJA1*) or did not change with age (*CTGF*) in the sheep. These observations suggest that these genes are not target genes for miR-133a in sheep or that there are additional contributors to cardiomyocyte quiescence that counter the effects of these pro-proliferative genes. Additional limitations associated with cross species comparative analyses of miRNA function are listed in the Methods section.

Although the miR-15 family has been implicated in regulation of cardiomyocyte proliferation in rodents [12, 27], there was remarkable variation in the expression profiles for the family members, being represented in Clusters 1, 2, 4 and 5. Since these family members have highly similar target mRNA, it is difficult to interpret the relevance of individual family members in regulating cardiomyocyte proliferation. One possibility is that the different expression patterns represent fine tuning of the ratio of this miRNA family to target mRNA at different development ages. The expression profiles of miR-15 family target mRNA (*CHEK1*, *CDC2A*, *BIRC5* and *SPAG5*) were consistent with decreased cardiomyocyte proliferation perhaps representing their specific roles in cell cycle regulation. The current findings in sheep however suggest that the miR-15 family may not be pivotal or direct regulators of the specific decrease in cardiomyocyte proliferation that occurs in late gestation and in early postnatal life in sheep.

While the exact role of each miRNA in Cluster 4, 1 and 3 is yet to be defined, it is noteworthy that miR-145, which has been implicated as an inhibitor of cell proliferation by down-regulating *IGF1R* in bladder cancer cells [44] and other cancers [45–47], was present in Cluster 4. Further analysis into the functional role of each miRNA identified in these clusters is required in order to determine which miRNA have a role in developmental cardiomyocyte quiescence.

### Identification of miRNA that may promote cardiomyocyte proliferation in sheep

In sheep, miRNA that have been previously identified as key promoters of proliferation in rodents are predicted to have decreased expression with age and therefore reside in Clusters 2, 5 or 6. miR-199a, which has been shown to stimulate proliferation in cultured rat cardiomyocytes [13] was identified in Cluster 2, however, further analysis using qRT-PCR demonstrated that miR-199a peaked around birth, then decreased into postnatal life to the level of that seen at 91 days gestation. Furthermore, the expression pattern of pro-proliferative miR-590 [13] did not allow its inclusion in any cluster and qRT-PCR demonstrated a similar expression at all ages. Considering the varied expression of their target genes, the outcomes of the present study suggest that miR-199a and miR-590 are not major regulators of developmental cardiomyocyte quiescence in sheep.

The roles of every miRNA in Clusters 2, 5 and 6 have not been identified, however, some miRNA in these clusters have been involved in aspects of the regulation of cell proliferation in other species. Recent studies have implicated the miR-17-92 group as an important regulator of cardiomyocyte proliferation and capable of stimulating previously quiescent, adult mice cardiomyocytes [48]. There are six members of the miR-17-92 group, which includes miR-17, miR-18a, miR-19a, miR-20a, miR-19b-1 and miR-92a-1. In the present study, miR-17 and miR-92 were in Cluster 2 (increased with age), which suggests a potential role of these miRNA in the inhibition of cardiomyocyte proliferation in late gestation, however, miR-20a and miR-19b were in Cluster 6 (peaked before birth, but then decreased into postnatal life) and the expression of miR-18a and 19a did not change with age. Thus, the specific roles of miRNA in the miR-17-92 group in regulating cardiomyocyte quiescence are unclear.

### The developmental regulation of an imprinted miRNA cluster during heart development

The present study has allowed us to isolate the period when most cardiomyocytes lose the ability to proliferate from other cellular processes, such as hypertrophy, that are upregulated after birth. Cluster 5 was particularly enriched for miRNA derived from an imprinted locus located on the telomeric end of OAR18 in sheep (Fig. 6). The syntenic region is conserved in placental mammals and it contains a large cluster of maternally expressed miRNA that may have complex roles in the cis- and trans-regulation of expression of genes within the broader imprinted ~1.1 Mb region or direct involvement in the maintenance of the imprinting status of the locus [39, 40, 49, 50]. A mutation (Callipyge) in this broader imprinted region in sheep strongly deregulates expression of multiple genes within the core of this locus, including the maternally expressed miRNA, and causes postnatal skeletal muscle hypertrophy in select muscles in a rostro-caudal gradient across the animal but only when the mutation is inherited in the context of the paternal heterozygote [39]. The mutation apparently does not impact cardiac function, although specific tests have not been undertaken to date. Whether the miRNA within this genomic locus are targeted at mRNA or involved in imprinting maintenance is not clear, but it is interesting to note that the miRNA from this cluster putatively target the KEGG *TGFβ pathway*. Multiple members of this pathway have strong roles in regulating muscle development [51] and cardiac development and disease [52, 53]. Maternally expressed genes typically limit aspects of offspring tissue growth and are often down-regulated after birth [54]. We therefore postulate that the down-regulation of these miRNA after birth may help coordinate the activation of negative regulators of growth thereby limiting cardiomyocyte size and possibly heart size relative to body size.

### Functional analysis of predicted targets of developmentally regulated miRNA clusters

The functional enrichment analyses for the predicted target genes for each miRNA cluster from the miRNA microarray study often showed enrichment for “*cancer*” related themes, which is likely indirectly reflecting proliferative potential, and also enrichment for the related terms *transcription factor regulation* and *proliferative activity.* Thus, there is a consistent functional theme relating to regulation of proliferation in the predicted target genes of the differentially expressed miRNA. One of the difficulties in the biological interpretation of miRNA data relates to the abundance of a miRNA, its mRNA targets and the effects of competing miRNA, which together interplay in a complex manner to regulate gene expression. Therefore, understanding the enriched biological themes in the current investigation will require functional modulation of multiple miRNA in an appropriate cell culture model of cardiomyocyte cell proliferation and differentiation, coupled with biochemical validation of putative miRNA target genes.

## Conclusion

This study has identified miRNA and their target genes associated with cardiac development across late gestation and into postnatal life in sheep, thereby isolating the transition of cardiomyocytes to quiescence that occurs in late gestation from the major physiological and biochemical changes that occur after birth. The investigation has identified a number of clusters of differentially expressed miRNA, some of which are consistent in timing with the suppression of cardiomyocyte proliferation, which occurs across the last third of gestation in sheep. The present study is also consistent with a coordinated and complex role of miRNA in the regulation of cardiomyocyte quiescence. The study identified substantial differences between sheep and rodent cardiac development that may reflect species differences underpinning the very different timing relative to birth of cardiomyocyte quiescence in these species. Importantly, through the use of miRNA microarrays, the current study highlights the potential involvement of many miRNA in the coordination of this pivotal developmental transition.

## Methods

All experiments were performed according to guidelines of both the University of Adelaide and the University of South Australia Animal Ethics Committees.

### Experimental protocol

#### Selection of gestational age groups

**Fig. 8:**
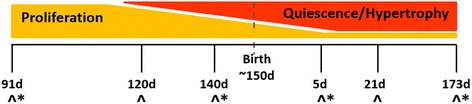
Timing of cardiomyocyte maturation in sheep and experimental design. Fetal and postnatal time points corresponding to the transition of cardiomyocytes from mononucleated and proliferative to quiescent and hypertrophic were chosen to analyse the expression of miRNA in the left ventricle by either qRT-PCR (^) or miRNA microarray (*)

In sheep before 100 days gestation (term, 150 ± 3 d gestation) all cardiomyocytes in the heart of the fetus are mononucleated and capable of proliferation, thereafter binucleation begins and hypertrophy becomes the predominant driver of cardiac growth [32, 33]. Therefore, we chose for analysis a gestational age when all cardiomyocytes were mononucleated (91 days; Fetal (F)90; n = 6; Fig. 8), gestational ages when the proportion of mononucleated cardiomyocytes will be decreasing (120 (F120) and 140 (F140) days; *n* = 8 and 6, respectively) and after birth when the majority of cardiomyocytes are quiescent, but undergoing metabolic maturation and hypertrophy (5 (postnatal (P)5), 21 (P21) and 173 (P173) days of age; *n* = 6, 6 and 9, respectively). For the expression of previously identified miRNA and target genes, quantitative real-time RT-PCR (qRT-PCR) was performed at each time point. For global miRNA expression, miRNA microarray analysis was performed using a subset of three animals from each of the following time points F91, F140, P5 and P173.

#### Tissue collection

For fetal time points (F21, F120 and F140), pregnant ewes were humanely killed with an overdose of sodium pentobarbitone (25 ml; 325 mg/mL; Virbac Aus, Peakhurst, Australia) and fetuses were delivered by hysterotomy, weighed and decapitated. For postnatal time points (P5, P21 and P173), lambs were humanely killed with an overdose of sodium pentobarbitone (as above). The heart was dissected, weighed and a consistent section of tissue from the free wall of the left ventricle was flash frozen in liquid nitrogen and stored at −80 °C for miRNA and mRNA analyses.

### Extraction of miRNA and mRNA

Total RNA from the left ventricle (~50 mg) was extracted using the miRNeasy Mini Kit (QIAGEN Pty Ltd, Doncaster, Australia). The miScript II RT Kit (QIAGEN Pty Ltd, Doncaster, Australia) with the miScript HiFlex Buffer was used to convert RNA into cDNA from 1 μg of purified RNA [55].

### Targeted measurement of miRNA and mRNA expression using qRT-PCR

#### miRNA expression

cDNA was synthesised using miScript II RT kit (QIAGEN Pty Ltd, Doncaster, Australia) following manufacturer’s instructions [55]. Controls containing no miScript II RT mix (NAC) and no RNA transcript (NTC) were used to test for genomic DNA and reagent contamination, respectively.

The reference miRNA (miR-26b, MS00009233; miR-92-1, MS00006594 and SNORD61-1, MS00033705, QIAGEN Pty Ltd, Doncaster, Australia)) were selected based on expression analysis using the geNorm component of the qBase relative quantification analysis software [56], because their expression was stable across samples [57]. The relative expression of miR-133a (MS00031423, QIAGEN Pty Ltd, Doncaster, Australia), the miR-15 family (miR-15a, MS00008785; miR-15b, MS00008799; miR-16, MS00031493; miR-195, MS00008953; miR-497, MS00031906; QIAGEN Pty Ltd, Doncaster, Australia), miR-199a (MS00007602; QIAGEN Pty Ltd, Doncaster, Australia) and miR-590 (MS00010269; QIAGEN Pty Ltd, Doncaster, Australia) were measured using quantitative real-time reverse transcription PCR (qRT-PCR) on an ABI ViiA7 (PE Applied Biosystems, Foster City, CA). miScript Primer Assays were used in conjunction with the miScript SYBR Green PCR Kit (QIAGEN Pty Ltd, Doncaster, Australia). Expression of each miRNA relative to the three stable reference miRNA was calculated using DataAssist Software 3.0 (PE Applied Biosystems, Foster City, CA) [56] and was expressed as mean normalised expression (MNE).

#### mRNA expression

cDNA was synthesized as previously described [58]. Controls containing no Superscript III (NAC) and no RNA transcript (NTC) were used to test for genomic DNA and reagent contamination, respectively.

The reference genes (ribosomal protein P0 [59], phosphoglycerate kinase 1 [60] and peptidylprolyl isomerase A [60]) were chosen based on expression analysis using the geNorm component of the qBase relative quantification analysis software [56, 61], because their expression was stable across samples [57]. The relative expression of mRNA transcripts of miRNA targets and the reference genes were measured by qRT-PCR using Fast SYBR® Green Master Mix (Applied Biosystems, CA, USA) in a final volume of 6 μL on a ViiA7 Fast Real-time PCR system (Applied Biosystems), as previously described [57].

Primers (Additional file 3) were validated as generating a single transcript and confirmed by the presence of one double stranded DNA product of the correct size and sequence. Controls containing no cDNA were included for each primer set on each plate to test for reagent contamination. Amplification efficiencies were determined from the slope of a plot of C_t_ (defined as the threshold cycle with the lowest significant increase in fluorescence) against the log of the cDNA template concentration (ranging from 1 to 100 ng). Expression of each transcript relative to the three stable reference genes was calculated using DataAssist Software 3.0 and was expressed as mean normalised expression (MNE).

#### Statistical analyses for miRNA and mRNA qRT-PCR

All data are presented as mean MNE ± SEM or as fold change from expression at 91 days gestation. A probability value of 5 % (*P* <0.05) was considered significant. Unless otherwise stated, comparisons between groups were analyzed for statistical significance with a one-way Analysis of Variance (ANOVA) followed by Duncans Multiple Comparison post hoc tests, where appropriate, using SPSS 20 for Windows (Statistical Package for Social Scientists Inc., IL, USA).

### miRNA microarray and data analysis

A custom designed miRNA microarray was employed using a service provider (LC Sciences, USA). It contained multiple (3–8) replicates of 3,098 unique probes and multiple replicates (8–80) of 56 control probes. The unique probes were derived from identified ovine miRNA and additional mammalian miRNA sequences downloaded from miRBase (http://www.mirbase.org/). The microarray included miRNA that have previously been shown to be involved in cardiomyocyte proliferation [7, 13, 27]. By its design the multispecies probe representation on the microarray resulted in miRNA redundancy. RNA, (2 μg) from three samples from each of the F91, F141, P5 and P173 groups, was used for analysis according to standard procedures (*e.g.* [62]). The experimental design included three biological replicates from each of the four developmental ages. Data were background corrected, log_2_ transformed and normalised using a procedure in the commercial package CLCBIO Genomics Workbench (http://www.clcbio.com/). ANOVA analysis performed in CLCBIO identified differentially expressed probes (FDR corrected *P* ≤ 0.05). Principal components analysis was also undertaken using the same package. Supplementary File 1 contains details of the differentially expressed probes. The microarray dataset has been deposited in the GEO database at NCBI (http://www.ncbi.nlm.nih.gov/geo/query/acc.cgi?acc=GSE68496).

#### k-means clusters

In k-means clustering, probes are clustered into k clusters for which the distances between probes within a cluster are small relative to the distance between clusters. The differentially expressed probes from the microarray were subjected to k-means clustering in CLCBIO using Euclidean distance and group means for the transformed expressed values for each developmental age. The difference between these values and the group mean across all ages for each probe was then determined to centre the data. A value of k = 6 was chosen as data elimination (25 %) revealed profile stability while k values greater than six did not produce substantially different profiles. Supplementary file 1 also contains cluster designations for the significantly differentially expressed probes.

#### miRNA target prediction and function analysis

A minimally redundant set of miRNA was derived from the probes in each k-means cluster and filtered for miRNA with mean expression across all samples less than 100. 3p and 5p miRNA arms were ignored as they cannot be used by most target prediction programs. miRNA target prediction can involve high false positive rates and hence conservative strategies are required. Consequently, the miRNA in each cluster were used as input into MiRWalk (http://www.umm.uni-heidelberg.de/apps/zmf/mirwalk/) for target prediction. MiRWalk used a consensus approach based on the intersection of at least seven of ten different methods and utilised target information from the human, rat and mouse genomes [42]. Redundant targets were then removed. Predicted targets were assessed for Gene Ontology (GO) term enrichments and KEGG pathway enrichments using DAVID (http://david.abcc.ncifcrf.gov/) [43]. Only terms with FDR corrected *P* < 0.05 were used. EASE scores were set at 0.05 to increase the analysis stringency.

#### Technical limitations of the study

To identify putative mRNA targets for the miRNA, a nonredundant set of miRNA corresponding to each k-means cluster was used for mRNA target prediction using MiRWalk. This strategy used a consensus of at least seven of ten different miRNA target prediction methods [42]. Enriched Gene Ontology (GO) terms were then identified using DAVID [43]. This conservative approach has three inherent assumptions: (i) the predicted mRNA targets are expressed in the investigated cardiac tissues; (ii) there is an inverse relationship between the direction of change in expression of a miRNA and its target mRNA, and; (iii) stoichiometry (ratio of abundance of miRNA to target) is not relevant.

There is a significant caveat when equating signal to abundance in the miRNA microarray analysis. The assumption is that the hybridization efficiency is constant. This may be unlikely for all probes, especially as it critically depends on GC content and probe length. Although both the sheep and mouse miRNA data are from miRNA microarrays using the same technologies, there may be species differences that result in small differences in sequence that affect hybridization. Further confounding this area is the nature of the probes, which in some cases relate to isomers.

## Additional files

Additional file 1:
**Statistics for differentially expressed probes.**
Additional file 2:
**DAVID KEGG pathway and GO functional enrichments for mRNA targets predicted from miRNA representing each k-means cluster.**
Additional file 3:
**Primer sequences used in Quantitative Real-Time Reverse Transcription-PCR.**

## Abbreviations

miRNA: microRNA
d: Days
RISC: RNA-induced silencing complex
CCND2: Cyclin D2
SRF: Serum response factor
GJA1: Connexin-43
PGAM1: Phosphoglycerate mutase
CTGF: Connective tissue growth factor
IGF1R: Insulin-like growth factor-1 receptor
Chek1: Checkpoint kinase 1
Cdk1/Cdc2a: Cyclin dependent kinase-1
BIRC5: Survivin/Baculoviral inhibitor of apoptosis repeat-containing 5
SPAG5: Sperm-associated antigen 5
CLIC5: Chloride intracellular channel protein 5
HOPX: Homeodomain-only protein
HOMER1: Homer protein homolog 1
qRT-PCR: Quantitative real-time RT-PCR
GO: Gene Ontology
CC: Cellular component
BP: Biological process
MF: Molecular function
MNE: Mean normalised expression
ANOVA: Analysis of variance
