# Supplementary material for: Regulation of microRNA during cardiomyocyte maturation in sheep
Source: BMC Genomics. 2015 Jul 22;16(1):541. doi: 10.1186/s12864-015-1693-z (PMC4509559; doi:10.1186/s12864-015-1693-z)
Supplement: Additional file 3: — Primer sequences used in Quantitative Real-Time Reverse Transcription-PCR. [file 12864_2015_1693_MOESM3_ESM.pdf]

Gene	Primers	Accession Number
Cyclin D2 (*CCND2*)	Fwd 5'- AGCACGCTCAGACCTTCATC -3'<br>Rev 5'- AGGCAATCCACATCCGTGTT -3'	NM_001127290.1
Serum response factor (*SRF*)	Fwd 5'- AGGCACTGATTCAGACCTGC -3'<br>Rev 5'- CAGACACTGGTGCCAGGTAG -3'	XM_004019222.1
Phosphoglyceric acid mutase (*PGAM1*)	Fwd 5'- TTGCACCCACTCCCTTCATACAGT -3'<br>Rev 5'- TCCTCATTGGTCACACCTCAGCAT -3'	NM_001034054
Connexin-43 (*GJA1*)	Fwd 5'- CTGGGTCCTGCAGATCATATTT -3'<br>Rev 5'- AGTTCCTCCTCTTTCTTGTTCAG -3'	XM_004011159.1
Connective tissue growth factor (*CTGF*)	Fwd 5'- TGCACCAGCATGAAGACATACCGA -3'<br>Rev 5'- ACAGGAAGTGTGGTGGTTCTGTGA -3'	NM_001164714.1
Insulin-like growth factor-1 receptor (*IGF1R*) [72]	Fwd 5'- AAGAACCATGCCTGCAGAAGG -3<br>Rev 5'- GGATTCTCAGGTTCTGGCCATT -3	
Checkpoint kinase 1 (*Chek1*)	Fwd 5'- CCATCCCAGACATCAAGAAAGA -3'<br>Rev 5'- TCCACCAGGAGACTCTGATAC -3'	XM_004019518.1
Cyclin dependent kinase-1 (*Cdk1*/*Cdc2a)*	Fwd 5'- ATGAGGTGGTTTGGCCAGGAGTTA -3'<br>Rev 5'- TGCGATAACAAGCTCCGTCCATCT -3'	NM_001142509.1
Survivin / baculoviral inhibitor of apoptosis repeat-containing 5 (*BIRC5)*	Fwd 5'- TTGAATCGCGGCACCGCTCG -3'<br>Rev 5'- GTGCAGGCGCAGCCCTCTAA -3'	NM_001001855.2
Sperm-associated antigen 5 (*SPAG5)*	Fwd 5'- TCCAGAGCCACAGAAACATC -3'<br>Rev 5'- GATGGACAGCAGAGTCTCATAAA -3'	XM_004012500.1
Chloride intracellular channel protein 5 (*CLIC5)*	Fwd 5'- CCTTCTCTCAGCGTCTCTTTATG -3'<br>Rev 5'- CAGGTCAGCTGGCTTTCTT -3'	XM_004018860.1
Homeodomain-only protein (*HOPX)*	Fwd 5'- CACTGTTAGCTGTCCTGCTATT -3'<br>Rev 5'- GCTGCACACAGCTTCCTAT -3'	NM_174097.2
Homer protein homolog 1 (*HOMER1)*	Fwd 5'- GCTCGACTAGCAAAGGAGAAA -3'<br>Rev 5'- CTGGTGTTAAAGGAGACTGAAGA -3'	NM_001076052.1
